# Dimethyl 2,6-dimethyl-1,4-dihydro­pyridine-3,5-dicarboxyl­ate

**DOI:** 10.1107/S1600536809035478

**Published:** 2009-09-09

**Authors:** Zhenfeng Zhang, Dong Xian, Jiange Wang, Guisheng Zhang

**Affiliations:** a College of Chemistry and Environmental Science, Henan Normal University, Xinxiang 453007, People’s Republic of China; b College of Chemistry, Luoyang Normal University, Xinxiang 453007, People’s Republic of China

## Abstract

In the crystal of the title compound, C_11_H_15_NO_4_, the mol­ecules are linked into sheets by N—H⋯O and C—H⋯O hydrogen bonds. Within the mol­ecule, the 1,4-dihydro­pyridine ring exhibits a distinctive planar conformation [r.m.s. deviation from the mean plane of 0.009 (3)Å], and the other non-H atoms are almost coplanar [r.m.s. deviation = 0.021 (3) Å] with the 1,4-dihydro­pyridine ring. The conformation of the latter is governed mainly by two intra­molecular C—H⋯O non-classical inter­actions.

## Related literature

For general background to the biological activity of 1,4-dihydro­pyridine derivatives, see: Kazda & Towart (1981 ▶); Janis & Triggle (1983 ▶); Núñez-Vergara *et al.*, (1998 ▶); Mak *et al.*, (2002 ▶). For their synthesis, see: Hantzsch & Liebigs (1882 ▶). For related structures, see: Bai *et al.* (2009 ▶); Quesada *et al.* (2006 ▶); Ramesh *et al.* (2008 ▶); Zhao & Teng (2008 ▶). For hydrogen-bond motifs, see: Bernstein *et al.* (1995 ▶).
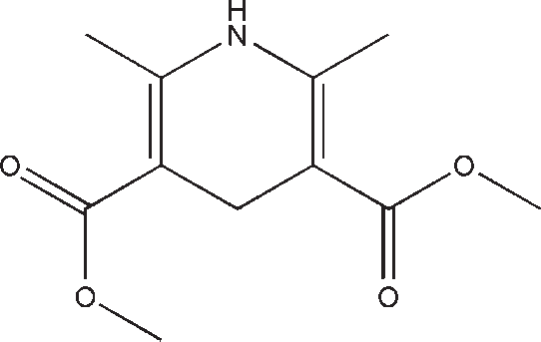


## Experimental

### 

#### Crystal data

C_11_H_15_NO_4_
*M* *_r_* = 225.24
Triclinic, 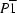

*a* = 7.3933 (13) Å
*b* = 7.8391 (14) Å
*c* = 11.1847 (19) Å
α = 75.977 (2)°
β = 75.274 (2)°
γ = 64.351 (2)°
*V* = 558.62 (17) Å^3^
*Z* = 2
Mo *K*α radiation
μ = 0.10 mm^−1^
*T* = 293 K
0.49 × 0.43 × 0.25 mm

#### Data collection

Bruker SMART CCD diffractometer
Absorption correction: multi-scan (*SADABS*; Sheldrick, 2003 ▶) *T*_min_ = 0.952, *T*_max_ = 0.965
3550 measured reflections
2047 independent reflections
1764 reflections with *I* > 2σ(*I*)
*R*_int_ = 0.015

#### Refinement

*R*[*F* ^2^ > 2σ(*F* ^2^)] = 0.040
*wR*(*F* ^2^) = 0.115
*S* = 1.05
2047 reflections
148 parameters
H-atom parameters constrained
Δρ_max_ = 0.16 e Å^−3^
Δρ_min_ = −0.23 e Å^−3^

### 

Data collection: *SMART* (Bruker, 1997 ▶); cell refinement: *SAINT* (Bruker, 1997 ▶); data reduction: *SAINT*; program(s) used to solve structure: *SHELXS97* (Sheldrick, 2008 ▶); program(s) used to refine structure: *SHELXL97* (Sheldrick, 2008 ▶); molecular graphics: *SHELXTL* (Sheldrick, 2008 ▶); software used to prepare material for publication: *SHELXTL*.

## Supplementary Material

Crystal structure: contains datablocks I, global. DOI: 10.1107/S1600536809035478/rk2162sup1.cif

Structure factors: contains datablocks I. DOI: 10.1107/S1600536809035478/rk2162Isup2.hkl

Additional supplementary materials:  crystallographic information; 3D view; checkCIF report

## Figures and Tables

**Table 1:** Hydrogen-bond geometry (Å, °)

*D*—H⋯*A*	*D*—H	H⋯*A*	*D*⋯*A*	*D*—H⋯*A*
N1—H1⋯O4^i^	0.86	2.15	3.006 (2)	176
C11—H11*B*⋯O2^ii^	0.96	2.60	3.219 (2)	122
C6—H6*D*⋯O2	0.96	2.09	2.843 (2)	134
C7—H7*D*⋯O3	0.96	1.98	2.733 (2)	134

Symmetry codes: (i) 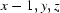; (ii) 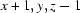.

**Fig. 1.**
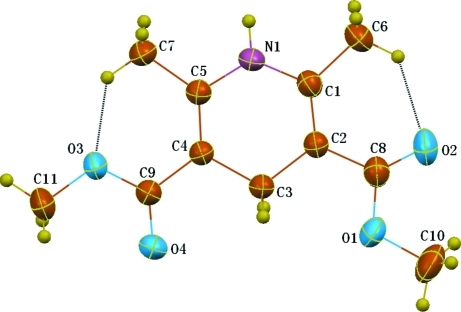
The molecular structure of I, showing the atom-labelling scheme. Displacement ellipsoids are drawn at the 30% probability level. H atoms are shown as small spheres of arbitrary radius. Only one component of the disordered methyl groups is shown.

**Fig. 2.**
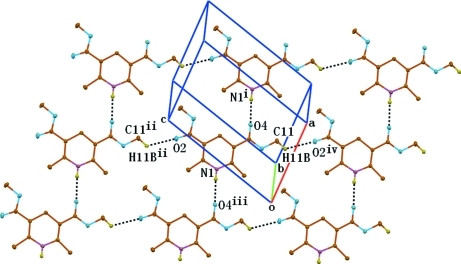
Part of the crystal structure of I, showing the formation of a (0 1 0) sheet. For the sake of clarity, H atoms not involved in the motif shown have been omitted. Intermolecular interactions are represented by dashed lines. Symmetry codes: (i) x+1, y, z; (ii) -x+1 ,y, z+1; (iii) x-1, y, z; (iv) x+1, y, z-1.

## supplementary crystallographic information

### Comment

The 1,4-dihydropyridine, (1,4-*DHP*) derivatives, as analogues of *NADH* coenzymes, exhibit a wide range of biological activities, acting as powerful arteriolar vasodilators (Kazda & Towart, 1981) and antihypertensives (Janis & Triggle, 1983). In addition, 1,4-*DHP* compounds such as nifedipine, nisoldipine and nicardipine exhibit potential trypanocidal activity (Núñez-Vergara *et al.*, 1998). The classical preparation method of 1,4-*DHP* is the Hantzsch (Hantzsch & Liebigs, 1882) and a number of 1,4-*DHP* derivatives have been synthesized *via* this method. We have prepared some 1,4-*DHP* derivatives by condensation reaction of β-enamino esters with aldehyde. As a typical example containing a planar 1,4-*DHP* ring, we now report the molecular and supramolecular structure of dimethyl 1,4-dihydro-2,6-dimethylpyridine-3,5-dicarboxylate, (I) (Fig. 1).

In the I, interestingly, 1,4-*DHP* ring exhibit perfectly coplanar conformation with r.m.s. deviation from the mean plane of 0.009 (3)Å. This conformation is significantly diverse from those found in other 1,4-*DHP* derivatives, where each of the 1,4-dihydropyrimidine rings adopts flat-boat conformation (Quesada *et al.*, 2006; Ramesh, *et al.*, 2008; Zhao & Teng, 2008; Bai *et al.*, 2009). Another point of interest in the conformation concerns the ester portion of the molecule. In each molecule, there are two short non-classical intramolecular C–H···O interactions (Table 1), and these, we think, control and stabilize the conformations of the two methoxycarbonyl fragments, which are both coplanar with the 1,4-*DHP* ring, as shown by the torsion angles. However, for C2-methoxycarbonyl it is carbonyl atom O2 that participates in the intramolecular hydrogen bond, and for C4-methoxycarbonyl it is ethoxy O3 atom. Within the 1,4-*DHP* ring, the C1-C2 and C4-C5 distances shows markedly two double bonds. The N1–C1 and N1–C5 bonds are significantly shorter than the standard N–C experimental bond length of 1.47Å (Mak, *et al.*, 2002). These features in bond distance suggest the existence of π-delocation in the C2/C1/N1/C5/C4 fragment.

Due to the above conformational features of I, its supramolecular structure exhibits some interesting feature. The molecules of the title compounds are linked into sheets by two independent intermolecular hydrogen bonds, one of N–H···O and one C–H···O type (Table 1), the formation of which is readily analyzed in terms of two one-dimensional substructures, one formed by the the N–H···O hydrogen bond and one formed by the C–H···O hydrogen bond. For the sake of simplicity, we shall omit any further consideration of other C–H···O intermolecular interaction involving C7-methyl group, which is too weak to influence the overall dimensionality of the supramolecular structure. In the first substructure, atom N1 in the molecule at (*x*, *y*, *z*) acts as a hydrogen-bond donor to the methoxycarbonyl atom O4 in the molecule at (*x*-1, *y*, *z*), thus forming by translation a C_2_^2^(6) (Bernstein *et al.*, 1995) chain running along the [1 0 0] direction (Fig. 2). In the second substructure, methyl atom C11 in the molecule at (*x*, *y*, *z*) acts as a hydrogen bond donor *via* H11B to methoxycarbonyl atom O2 in the milecule at (*x*+1, *y*, *z*-1), so forming by translation a C(9) (Bernstein *et al.*, 1995) chain parallel to the [-1 0 1] direction (Fig. 2). The combination of the two chain motifs is sufficient to link all the molecules into a two-dimensional sheet parallel to (0 1 0). Two such sheets pass through each unit cell in the domains 0 < *y* < 1/2 and 1/2 < *y* < 1, and there are no direction-specific interactions between the two sheets.

### Experimental

Into a three-necked round-bottomed flask equipped with a stirrer were introduced methyl 3-aminobut-2-enoate (0.1 mol, 11.5 g), aqueous formaldehyde (0.05 mol, 37% 4.0 g) and ethanol (95%, 25 ml). The resulted mixture was refluxed with stirring for *ca* 20 min, and then the solution is cooled to room temperature. The precipitate was filtered off, washed with cool ethanol (95%), and the resulting solid product was recrystallized from hot ethanol to give crystals of I.

^1^H NMR (*DMSO*, 400 MHz) of (I): δ 8.35 (s, 1H), δ 3.59 (s, 6H), δ 3.14 (s, 2H), δ 2.12 (s, 6H).

### Refinement

All H atoms other than the C1- and C5-methyl H atoms were located in a difference map and then treated as riding atoms with C–H distances of 0.96Å (CH_3_) or 0.97Å (CH_2_), and N–H distance of 0.86Å with *U*_iso_(H) = 1.2*U*_eq_(C, N) or 1.5*U*_eq_(methyl C). The C1- and C5-methyl H atoms was modelled as idealized disordered methyl groups over two sets offset by 60°.

### Crystal data

**Table:**

C_11_H_15_NO_4_	*Z* = 2
*M**_r_* = 225.24	*F*(000) = 240
Triclinic, *P*1	*D*_x_ = 1.339 Mg m^−^^3^
Hall symbol: -P 1	Mo *K*α radiation, λ = 0.71073 Å
*a* = 7.3933 (13) Å	Cell parameters from 2071 reflections
*b* = 7.8391 (14) Å	θ = 2.9–28.1°
*c* = 11.1847 (19) Å	µ = 0.10 mm^−^^1^
α = 75.977 (2)°	*T* = 293 K
β = 75.274 (2)°	Block, blue
γ = 64.351 (2)°	0.49 × 0.43 × 0.25 mm
*V* = 558.62 (17) Å^3^	

### Data collection

**Table:**

Bruker SMART CCD diffractometer	2047 independent reflections
Radiation source: fine-focus sealed tube	1764 reflections with *I* > 2σ(*I*)
graphite	*R*_int_ = 0.015
φ and ω scans	θ_max_ = 25.5°, θ_min_ = 2.9°
Absorption correction: multi-scan (*SADABS*; Sheldrick, 2003)	*h* = −8→8
*T*_min_ = 0.952, *T*_max_ = 0.965	*k* = −9→9
3550 measured reflections	*l* = −12→13

### Refinement

**Table:**

Refinement on *F*^2^	Secondary atom site location: difference Fourier map
Least-squares matrix: full	Hydrogen site location: inferred from neighbouring sites
*R*[*F*^2^ > 2σ(*F*^2^)] = 0.040	H-atom parameters constrained
*wR*(*F*^2^) = 0.115	*w* = 1/[σ^2^(*F*_o_^2^) + (0.0612*P*)^2^ + 0.1212*P*] where *P* = (*F*_o_^2^ + 2*F*_c_^2^)/3
*S* = 1.05	(Δ/σ)_max_ < 0.001
2047 reflections	Δρ_max_ = 0.16 e Å^−^^3^
148 parameters	Δρ_min_ = −0.23 e Å^−^^3^
0 restraints	Extinction correction: *SHELXL97* (Sheldrick, 2008), Fc^*^=kFc[1+0.001xFc^2^λ^3^/sin(2θ)]^-1/4^
Primary atom site location: structure-invariant direct methods	Extinction coefficient: 0.26 (2)

### Special details

**Table:**

Geometry. All s.u.'s (except the s.u. in the dihedral angle between two l.s. planes) are estimated using the full covariance matrix. The cell s.u.'s are taken into account individually in the estimation of s.u.'s in distances, angles and torsion angles; correlations between s.u.'s in cell parameters are only used when they are defined by crystal symmetry. An approximate (isotropic) treatment of cell s.u.'s is used for estimating s.u.'s involving l.s. planes.
Refinement. Refinement of *F*^2^ against ALL reflections. The weighted *R*-factor *wR* and goodness of fit *S* are based on *F*^2^, conventional *R*-factors *R* are based on *F*, with *F* set to zero for negative *F*^2^. The threshold expression of *F*^2^ > σ(*F*^2^) is used only for calculating *R*-factors(gt) *etc*. and is not relevant to the choice of reflections for refinement. *R*-factors based on *F*^2^ are statistically about twice as large as those based on *F*, and *R*-factors based on ALL data will be even larger.

### Fractional atomic coordinates and isotropic or equivalent isotropic displacement parameters (Å^2^)

**Table:**

	*x*	*y*	*z*	*U*_iso_*/*U*_eq_	Occ. (<1)
C1	−0.2092 (2)	0.24631 (19)	0.60198 (13)	0.0371 (3)	
C2	−0.0517 (2)	0.24220 (18)	0.64524 (13)	0.0355 (3)	
C3	0.1441 (2)	0.2394 (2)	0.55870 (13)	0.0366 (3)	
H3A	0.1702	0.3463	0.5677	0.044*	
H3B	0.2561	0.1224	0.5837	0.044*	
C4	0.13729 (19)	0.25116 (18)	0.42266 (12)	0.0328 (3)	
C5	−0.0262 (2)	0.25369 (18)	0.38665 (12)	0.0348 (3)	
C6	−0.4103 (2)	0.2499 (3)	0.67563 (16)	0.0519 (4)	
H6A	−0.4925	0.2528	0.6204	0.078*	0.50
H6B	−0.4787	0.3618	0.7161	0.078*	0.50
H6C	−0.3888	0.1374	0.7376	0.078*	0.50
H6D	−0.4142	0.2485	0.7623	0.078*	0.50
H6E	−0.4280	0.1396	0.6666	0.078*	0.50
H6F	−0.5178	0.3639	0.6452	0.078*	0.50
C7	−0.0548 (2)	0.2638 (2)	0.25662 (14)	0.0467 (4)	
H7A	−0.1855	0.2633	0.2596	0.070*	0.50
H7B	0.0502	0.1553	0.2209	0.070*	0.50
H7C	−0.0474	0.3794	0.2062	0.070*	0.50
H7D	0.0637	0.2687	0.1982	0.070*	0.50
H7E	−0.1720	0.3767	0.2369	0.070*	0.50
H7F	−0.0744	0.1526	0.2516	0.070*	0.50
C8	−0.0642 (2)	0.2376 (2)	0.77872 (14)	0.0430 (4)	
C9	0.3211 (2)	0.25935 (19)	0.33790 (13)	0.0359 (3)	
C10	0.1095 (4)	0.2341 (3)	0.93016 (16)	0.0699 (6)	
H10A	−0.0084	0.3384	0.9621	0.105*	
H10B	0.2303	0.2470	0.9358	0.105*	
H10C	0.1084	0.1152	0.9784	0.105*	
C11	0.5033 (3)	0.2757 (3)	0.13100 (16)	0.0647 (5)	
H11A	0.5180	0.3911	0.1320	0.097*	
H11B	0.4925	0.2720	0.0480	0.097*	
H11C	0.6199	0.1669	0.1563	0.097*	
N1	−0.19256 (17)	0.24966 (18)	0.47535 (11)	0.0401 (3)	
H1	−0.2931	0.2492	0.4504	0.048*	
O1	0.10679 (18)	0.23698 (18)	0.80131 (9)	0.0537 (3)	
O2	−0.2029 (2)	0.2326 (2)	0.86306 (11)	0.0705 (4)	
O3	0.32231 (16)	0.27138 (19)	0.21605 (10)	0.0547 (3)	
O4	0.46494 (15)	0.25501 (17)	0.37415 (10)	0.0499 (3)	

### Atomic displacement parameters (Å^2^)

**Table:**

	*U*^11^	*U*^22^	*U*^33^	*U*^12^	*U*^13^	*U*^23^
C1	0.0345 (7)	0.0403 (7)	0.0361 (7)	−0.0168 (6)	−0.0018 (6)	−0.0055 (5)
C2	0.0353 (7)	0.0396 (7)	0.0318 (7)	−0.0161 (6)	−0.0029 (5)	−0.0060 (5)
C3	0.0323 (7)	0.0479 (8)	0.0329 (7)	−0.0183 (6)	−0.0047 (5)	−0.0082 (6)
C4	0.0314 (7)	0.0376 (7)	0.0307 (7)	−0.0148 (5)	−0.0047 (5)	−0.0062 (5)
C5	0.0341 (7)	0.0384 (7)	0.0338 (7)	−0.0161 (5)	−0.0059 (5)	−0.0052 (5)
C6	0.0397 (8)	0.0718 (11)	0.0470 (9)	−0.0287 (7)	0.0017 (7)	−0.0108 (7)
C7	0.0443 (8)	0.0679 (10)	0.0366 (8)	−0.0290 (7)	−0.0102 (6)	−0.0063 (7)
C8	0.0458 (8)	0.0471 (8)	0.0353 (8)	−0.0201 (6)	−0.0011 (6)	−0.0077 (6)
C9	0.0329 (7)	0.0415 (7)	0.0341 (7)	−0.0157 (6)	−0.0052 (5)	−0.0058 (5)
C10	0.0973 (15)	0.0926 (14)	0.0354 (9)	−0.0492 (12)	−0.0171 (9)	−0.0088 (8)
C11	0.0512 (10)	0.1089 (15)	0.0386 (9)	−0.0428 (10)	0.0079 (7)	−0.0144 (9)
N1	0.0324 (6)	0.0575 (7)	0.0369 (7)	−0.0239 (5)	−0.0061 (5)	−0.0066 (5)
O1	0.0599 (7)	0.0802 (8)	0.0319 (6)	−0.0359 (6)	−0.0090 (5)	−0.0106 (5)
O2	0.0668 (8)	0.1125 (11)	0.0357 (6)	−0.0465 (8)	0.0095 (6)	−0.0153 (6)
O3	0.0457 (6)	0.0966 (9)	0.0316 (6)	−0.0411 (6)	0.0011 (4)	−0.0101 (5)
O4	0.0356 (6)	0.0772 (8)	0.0430 (6)	−0.0284 (5)	−0.0049 (4)	−0.0103 (5)

### Geometric parameters (Å, °)

**Table:**

C1—C2	1.356 (2)	C7—H7B	0.9600
C1—N1	1.3848 (18)	C7—H7C	0.9600
C1—C6	1.4976 (19)	C7—H7D	0.9600
C2—C8	1.465 (2)	C7—H7E	0.9600
C2—C3	1.5172 (18)	C7—H7F	0.9600
C3—C4	1.5142 (18)	C8—O2	1.2114 (19)
C3—H3A	0.9700	C8—O1	1.3489 (19)
C3—H3B	0.9700	C9—O4	1.2154 (17)
C4—C5	1.3587 (19)	C9—O3	1.3411 (17)
C4—C9	1.4634 (18)	C10—O1	1.4410 (19)
C5—N1	1.3771 (17)	C10—H10A	0.9600
C5—C7	1.5007 (19)	C10—H10B	0.9600
C6—H6A	0.9600	C10—H10C	0.9600
C6—H6B	0.9600	C11—O3	1.4396 (18)
C6—H6C	0.9600	C11—H11A	0.9600
C6—H6D	0.9600	C11—H11B	0.9600
C6—H6E	0.9600	C11—H11C	0.9600
C6—H6F	0.9600	N1—H1	0.8600
C7—H7A	0.9600		
			
C2—C1—N1	119.37 (12)	C5—C7—H7C	109.5
C2—C1—C6	127.69 (13)	H7A—C7—H7C	109.5
N1—C1—C6	112.93 (12)	H7B—C7—H7C	109.5
C1—C2—C8	120.67 (12)	C5—C7—H7D	109.5
C1—C2—C3	121.77 (12)	H7A—C7—H7D	141.1
C8—C2—C3	117.56 (12)	H7B—C7—H7D	56.3
C4—C3—C2	112.94 (11)	H7C—C7—H7D	56.3
C4—C3—H3A	109.0	C5—C7—H7E	109.5
C2—C3—H3A	109.0	H7A—C7—H7E	56.3
C4—C3—H3B	109.0	H7B—C7—H7E	141.1
C2—C3—H3B	109.0	H7C—C7—H7E	56.3
H3A—C3—H3B	107.8	H7D—C7—H7E	109.5
C5—C4—C9	124.99 (12)	C5—C7—H7F	109.5
C5—C4—C3	121.76 (12)	H7A—C7—H7F	56.3
C9—C4—C3	113.25 (11)	H7B—C7—H7F	56.3
C4—C5—N1	119.50 (12)	H7C—C7—H7F	141.1
C4—C5—C7	127.93 (12)	H7D—C7—H7F	109.5
N1—C5—C7	112.57 (11)	H7E—C7—H7F	109.5
C1—C6—H6A	109.5	O2—C8—O1	121.07 (14)
C1—C6—H6B	109.5	O2—C8—C2	127.86 (15)
H6A—C6—H6B	109.5	O1—C8—C2	111.06 (12)
C1—C6—H6C	109.5	O4—C9—O3	121.45 (12)
H6A—C6—H6C	109.5	O4—C9—C4	122.90 (13)
H6B—C6—H6C	109.5	O3—C9—C4	115.65 (11)
C1—C6—H6D	109.5	O1—C10—H10A	109.5
H6A—C6—H6D	141.1	O1—C10—H10B	109.5
H6B—C6—H6D	56.3	H10A—C10—H10B	109.5
H6C—C6—H6D	56.3	O1—C10—H10C	109.5
C1—C6—H6E	109.5	H10A—C10—H10C	109.5
H6A—C6—H6E	56.3	H10B—C10—H10C	109.5
H6B—C6—H6E	141.1	O3—C11—H11A	109.5
H6C—C6—H6E	56.3	O3—C11—H11B	109.5
H6D—C6—H6E	109.5	H11A—C11—H11B	109.5
C1—C6—H6F	109.5	O3—C11—H11C	109.5
H6A—C6—H6F	56.3	H11A—C11—H11C	109.5
H6B—C6—H6F	56.3	H11B—C11—H11C	109.5
H6C—C6—H6F	141.1	C5—N1—C1	124.57 (11)
H6D—C6—H6F	109.5	C5—N1—H1	117.7
H6E—C6—H6F	109.5	C1—N1—H1	117.7
C5—C7—H7A	109.5	C8—O1—C10	115.57 (13)
C5—C7—H7B	109.5	C9—O3—C11	116.70 (12)
H7A—C7—H7B	109.5		
			
N1—C1—C2—C8	−179.68 (12)	C1—C2—C8—O1	−179.05 (12)
C6—C1—C2—C8	1.0 (2)	C3—C2—C8—O1	1.79 (18)
N1—C1—C2—C3	−0.6 (2)	C5—C4—C9—O4	−179.32 (13)
C6—C1—C2—C3	−179.85 (13)	C3—C4—C9—O4	0.73 (19)
C1—C2—C3—C4	2.74 (19)	C5—C4—C9—O3	0.4 (2)
C8—C2—C3—C4	−178.11 (11)	C3—C4—C9—O3	−179.52 (11)
C2—C3—C4—C5	−3.18 (18)	C4—C5—N1—C1	1.1 (2)
C2—C3—C4—C9	176.77 (11)	C7—C5—N1—C1	−177.92 (12)
C9—C4—C5—N1	−178.51 (12)	C2—C1—N1—C5	−1.6 (2)
C3—C4—C5—N1	1.4 (2)	C6—C1—N1—C5	177.80 (12)
C9—C4—C5—C7	0.4 (2)	O2—C8—O1—C10	−1.3 (2)
C3—C4—C5—C7	−179.65 (13)	C2—C8—O1—C10	179.51 (13)
C1—C2—C8—O2	1.8 (2)	O4—C9—O3—C11	1.0 (2)
C3—C2—C8—O2	−177.38 (15)	C4—C9—O3—C11	−178.77 (14)

### Hydrogen-bond geometry (Å, °)

**Table:**

*D*—H···*A*	*D*—H	H···*A*	*D*···*A*	*D*—H···*A*
N1—H1···O4^i^	0.86	2.15	3.006 (2)	176
C11—H11B···O2^ii^	0.96	2.60	3.219 (2)	122
C6—H6D···O2	0.96	2.09	2.843 (2)	134
C7—H7D···O3	0.96	1.98	2.733 (2)	134

Symmetry codes: (i) *x*−1, *y*, *z*; (ii) *x*+1, *y*, *z*−1.
